# How brain-computer interface technology may improve the diagnosis of the disorders of consciousness: A comparative study

**DOI:** 10.3389/fnins.2022.959339

**Published:** 2022-08-11

**Authors:** Rossella Spataro, Yiyan Xu, Ren Xu, Giorgio Mandalà, Brendan Z. Allison, Rupert Ortner, Alexander Heilinger, Vincenzo La Bella, Christoph Guger

**Affiliations:** ^1^IRCCS Centro Neurolesi Bonino Pulejo, Palermo, Italy; ^2^ALS Clinical Research Center, University of Palermo, Palermo, Italy; ^3^g.tec Medical Engineering GmbH, Schiedlberg, Austria; ^4^Rehabilitation Unit, Ospedale Buccheri La Ferla, Palermo, Italy; ^5^Cognitive Science Department, University of California, San Diego, San Diego, United States; ^6^g.tec Medical Engineering Spain S.L., Barcelona, Spain

**Keywords:** brain-computer interface, Coma Recovery Scale-Revised, covert consciousness, unresponsive wakefulness syndrome, minimally conscious state, vegetative state

## Abstract

**Objective:**

Clinical assessment of consciousness relies on behavioural assessments, which have several limitations. Hence, disorder of consciousness (DOC) patients are often misdiagnosed. In this work, we aimed to compare the repetitive assessment of consciousness performed with a clinical behavioural and a Brain-Computer Interface (BCI) approach.

**Materials and methods:**

For 7 weeks, sixteen DOC patients participated in weekly evaluations using both the Coma Recovery Scale-Revised (CRS-R) and a vibrotactile P300 BCI paradigm. To use the BCI, patients had to perform an active mental task that required detecting specific stimuli while ignoring other stimuli. We analysed the reliability and the efficacy in the detection of command following resulting from the two methodologies.

**Results:**

Over repetitive administrations, the BCI paradigm detected command following before the CRS-R in seven patients. Four clinically unresponsive patients consistently showed command following during the BCI assessments.

**Conclusion:**

Brain-Computer Interface active paradigms might contribute to the evaluation of the level of consciousness, increasing the diagnostic precision of the clinical bedside approach.

**Significance:**

The integration of different diagnostic methods leads to a better knowledge and care for the DOC.

## Introduction

The diagnosis of disorders of consciousness (DOCs) and locked-in syndrome (LIS) still represents a clinical challenge. The most severe type of DOC is Coma, characterised by closed eyes and no volitional responses to commands or sensory stimulation. Unresponsive Wakefulness Syndrome (UWS) is a slightly less severe state in which eye-opening appears without other changes in responsivity. Consistent voluntary reactions to external stimuli characterise the Minimal Conscious State (MCS), whereas a functional use of objects or accurate communication denotes the Emergence from the MCS (EMCS). In the Complete Locked-in Syndrome (CLIS), no voluntary movements are possible and thus communication is impossible with mainstream technologies, even if the patient has intact cognitive functioning (Giacino et al., 2014).

Evaluating the cognitive capabilities and conscious functions of patients who cannot communicate nor show volitional behaviours is very difficult at the bedside. The resulting diagnostic errors have major ethical implications. The families of people suffering from DOCs must make difficult decisions about ongoing medical care, treatment or rehabilitation options, housing, visitation, and even the withdrawal of life support. These families and patients need accurate information about the patient’s remaining conscious function and likelihood of recovery (Young and Schiff, 2014).

In mainstream medical practice, DOCs are universally diagnosed through clinical consensus and standardised scales (Giacino et al., 2018). The Coma Recovery Scale-Revised (CRS-R, Giacino et al., 2004) is the most validated tool, with good or excellent content validity, interrater reliability and internal consistency (Seel et al., 2010). However, many patients cannot generate reproducible responses to the CRS-R due to fluctuations in the level of arousal, motor impairment, severe hypertonus and other clinical characteristics. Moreover, the CRS-R administration needs qualified assessors and is time consuming, so that in many cases it is replaced by other diagnostic tools or a “judgment call” (Formisano et al., 2019). However, it has been estimated that up to 43% of patients judged as unresponsive by clinical consensus have minimal consciousness (Andrews, 1996; Schnakers et al., 2009).

Different functional imaging paradigms have been developed to improve the accuracy of DOC diagnosis. For instance, performing spatial and motor imagery mental tasks (Monti et al., 2010) and word recognition tasks (Monti et al., 2015) in an fMRI revealed covert (not evident from overt behaviour) command following in a small proportion of UWS patients. However, many target patients can’t be assessed with fMRI because of technical issues, such as spontaneous movements or metallic implants. Moreover, in a comparison study with 18F-FDG-PET, the fMRI results yielded unsatisfying sensitivity to the MCS, low congruence with the CRS-R and weak outcome prediction power (Stender et al., 2014). In the same and other studies (Bodart et al., 2017), the 18F-FDG-PET showed the potential to detect preserved metabolism in behaviourally unresponsive subjects, with very high sensitivity to the MCS and a reliable negative prediction of recovery of consciousness at 1 year.

Despite possible benefits, such sophisticated neuroimaging approaches are relatively expensive, require highly specialised skills, and entail moving the patient. Consequently, most DOC patients admitted to clinical centres in the near future are unlikely to be assessed with these methodologies.

EEG-based assessments do not require moving the patient from the bed nor complex equipment and could be performed in any hospital ward. Several passive and active EEG paradigms have been explored to complement the behavioural evaluation of consciousness. In passive paradigms, the brain activity passively elicited by external stimuli is recorded and analysed. Conversely, active EEG paradigms use a willful mental task to generate brain activity, such as listening to the user’s own and other names, mental counting of specific stimuli, or motor imagery (Owen et al., 2006; Goldfine et al., 2011; Claassen et al., 2019). The recognition of a rare stimulus amidst a series of frequent stimuli, which is the basic principle of the classic “oddball paradigm,” evokes event-related potential markers of bottom-up attention, or P3a (Polich, 2007). Conversely, the discrimination of target and non-target stimuli of equal frequency denotes top-down attentional effects related to a task-relevant P3b component (Gibson et al., 2016).

Systems using EEG-based brain-computer interface (BCI) approaches have been employed to detect covert awareness and command following in patients who could not otherwise communicate. For instance, a system based on the P300 somatosensory evoked potential revealed the volitional execution of a mental task in two of three CLIS patients, nine of twelve LIS patients (Guger et al., 2017) and five of twelve UWS patients (Guger et al., 2018). Using an audio-visual P300 paradigm, Wang et al. (2017) collected responsive results in four out of eight UWS patients, whereas Pan et al. (2018) reported the detection of emotion recognition and covert command following in one out of three UWS patients using a visual P300-based BCI approach. Furthermore, classification accuracy *via* somatosensory (Spataro et al., 2018) and visual (Pan et al., 2020) P300 BCI approaches could help predict of the recovery of behavioural responses in the following 3–6 months.

However, most of these studies are based on a single assessment. Since DOC patients may exhibit highly variable performance in BCI paradigms both within and across sessions, single assessments may cause misleading results (Allison et al., 2020).

In this study, we compared the efficiency of the CRS-R and a vibrotactile P300 BCI-based paradigm in the detection of command following across multiple assessment sessions.

## Materials and methods

### Participants

We initially enrolled a sample of eighteen consecutive DOCs patients at the time of the admission to the Rehabilitation Unit of Buccheri La Ferla Hospital (R1) and the IRCCS Centro Neurolesi Bonino Pulejo (R2). Before the recruitment, we presented the scope, risks and limits of the study to the legal representative of each patient, and after a comprehensive disclosure, we obtained written informed consent from sixteen of them. The Ethics Committee of the IRCCS Centro Neurolesi Bonino Pulejo approved the study.

Table 1 shows the patients’ clinical and demographic characteristics. Eight patients were suffering from the consequences of a traumatic brain injury. In the remaining eight cases, the DOC was a result of other acute neurological disorders (ischaemic stroke, encephalitis, cerebral haemorrhage, hypoxia-ischemia brain injury). The mean age was 35.8 ± 16.5 (range: 16–70) years, and the mean disease duration was 28.6 ± 39 (range: 2–150) months. As is expected in a rehabilitation environment, all the patients were in stable clinical conditions, without the need for sedative drugs or intensive care. Two patients suffered from chronic respiratory insufficiency and received long-term mechanical ventilation treatment. Throughout the study period, each patient’s medical interventions and any rehabilitation plans continued without modifications due to this study.

**TABLE 1:** Clinical and demographic characteristics of the recruited patients.

Patient	Centre	Sex	Age (years)	Aetiology	Disease duration (months)	Mechanical ventilation
Patient 1	R1	F	39	CH	8	No
Patient 2	R1	F	38	TBI	13	No
Patient 3	R1	F	22	TBI	2	No
Patient 4	R1	F	30	ENC	4	Yes
Patient 5	R2	M	26	TBI	13	No
Patient 6	R2	F	18	TBI	4	Yes
Patient 7	R2	M	16	CH	11	No
Patient 8	R2	M	20	TBI	24	No
Patient 9	R2	M	41	TBI	6	No
Patient 10	R2	M	60	IS	150	No
Patient 11	R2	M	25	TBI	7	No
Patient 12	R2	F	59	IS	60	No
Patient 13	R2	M	70	IS	2	No
Patient 14	R2	M	23	TBI	34	No
Patient 15	R2	M	37	HBI	44	No
Patient 16	R2	M	64	HBI	59	No

R1, Rehabilitation Unit of Buccheri La Ferla Hospital. R2, Rehabilitation Unit of IRCCS Centro Neurolesi Bonino Pulejo. CH, Cerebral Haemorrhage; TBI, Traumatic Brain Injury; ENC, encephalitis; IS, ischemic stroke; HBI, Hypoxia-Ischemia Brain Injury.

### Neurobehavioural assessment

An experienced neurologist (RS) administered the CRS-R to discriminate the level of consciousness. The scale consists of six subscales exploring auditory, visual and motor functions, as well as the arousal state and the communication function. Eye opening (spontaneous or after stimulation) without evidence of volitional responses to the stimuli leads to the diagnosis of UWS, whereas the execution of verbal commands, the visual fixation or pursuit, an intentional communication or the localisation of noxious stimuli denote an MCS. This group of patients is further subcategorized as MCS plus or MCS minus on the basis of the evidence in the first group of language processing, expressed by command following or communication (Bruno et al., 2012). An accurate communication or functional use of objects denotes the EMCS.

The CRS-R administration lasted approximately 20 min and was repeated weekly for 7 weeks. We scheduled the administration for each patient at the same time. If the patient was sleeping, we postponed the evaluation to the best time in the same day. When needed, we applied the CRS-R Arousal Facilitation Protocol.

### Brain-Computer Interface-based assessment

All BCI-based sessions were conducted blindly by two experimenters (YX and AH), without access to the clinical file nor any CRS-R assessment results.

**FIGURE 1:**
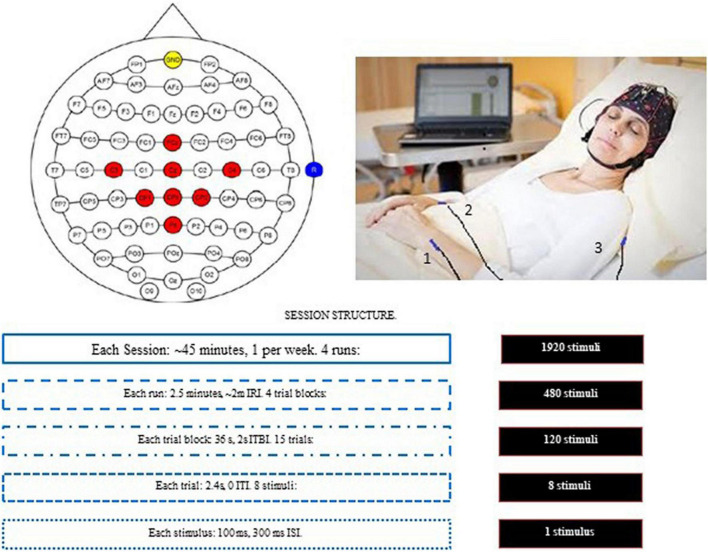
The experimental paradigm. Top left: electrode montage. The red spots mark the positions of eight active EEG electrodes. The reference was placed on the right earlobe (blue) and the ground electrode was at FPz (yellow). Top right: the position of the three vibrotactile stimulators: 1 (left wrist); 2 (right wrist); and 3 (back). Each trial had eight stimuli: six were to vibrotactile stimulator 3, and 1 (each) to vibrotactile stimulators 1 and 2. Bottom: The components within each session. IRI, inter-run interval; ITBI, Inter-trial block interval; ITI, Inter-trial interval; ISI, Inter-stimulus interval. The boxes on the right show the number of stimuli for that row.

On the same days as each CRS-R administration, after at least 2 h of rest, each patient participated in a BCI-based session using the mindBEAGLE system (g.tec medical engineering GmbH, Austria). This system, previously validated with healthy subjects and different patient groups (Guger et al., 2017, 2018; Spataro et al., 2018) includes a laptop, an EEG amplifier (g.USBamp), a cap with 8 wet active electrodes and three vibrotactile stimulators. While the patient was in a comfortable seated or supine position, the cap was placed safely on the scalp, the left and right stimulators were gently fixed on the corresponding wrists, and a third (distractor) stimulator was fixed on the back (Figure 1).

Each session contained four runs, with a break of about 2 min between runs. Each session required about 45 min, including preparation and de-prep. Each run lasted about 2.5 min and contained four trial blocks. At the beginning of each trial block, patients heard a verbal instruction from the system asking them to silently count stimuli to either the left or right wrist. 50% of the trial blocks (determined pseudorandomly) designated the right wrist as the target. The experimenter also touched the patient on the left or right arm to provide instruction to silently count vibrations on the corresponding wrist. This was meant as a backup measure to the verbal instruction to counteract possible impairments due to attention, language comprehension or other deficits.

Each trial block consisted of 30 trials. Each trial contained eight stimuli (100 ms each, 400 ms between stimulus onset): one stimulation of each wrist and six distractor stimuli, in pseudorandom order. Throughout this paper, “non-target stimuli” refers to both distractor stimuli (to the back) and stimuli to the non-target wrist (either right or left). Thus, each trial contained one target stimulus and seven non-target stimuli.

### Signal processing and classification

During each run, the system recorded the raw EEG data and each stimulation onset and trained a classifier which discriminated targets from non-targets for every subject accordingly. The acquired EEG data were bandpass filtered between 0.1 and 30 Hz to remove baseline shifts and eliminate most EMG artefacts.

**FIGURE 2:**
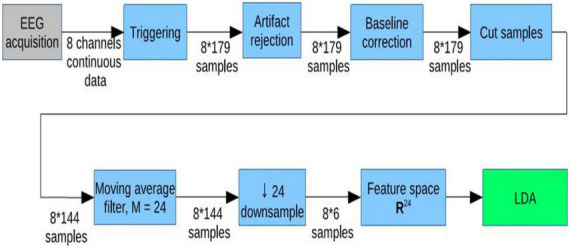
Signal processing steps. * Means multiply (same as x).

The target and non-target stimuli were randomly assigned into two equal sized pools. One pool was used to train a classifier, and the other pool was used to test the classifier. The data were classified using linear discriminant analysis (LDA) to distinguish the target from non-target stimuli. Figure 2 depicts the signal processing steps. Data segments of −100 to 600 ms around each stimulus were extracted and baseline corrected. In the next step, samples containing the baseline information were removed from the single trials. To reduce the dimensionality of feature space, a downsampling by factor 24 was done on the remaining trials. Since the downsampling decreases the Nyquist frequency, a moving average filter had been applied beforehand, to prevent aliasing. What remained were six samples in each of the eight channels, containing amplitude values of the ERP. These eight times six samples generated the feature space that is used for the LDA classification. A 10-fold cross validation was used. This results in a classification accuracy ranging from 0 to 100% that describes how well the data can be separated.

**FIGURE 3:**
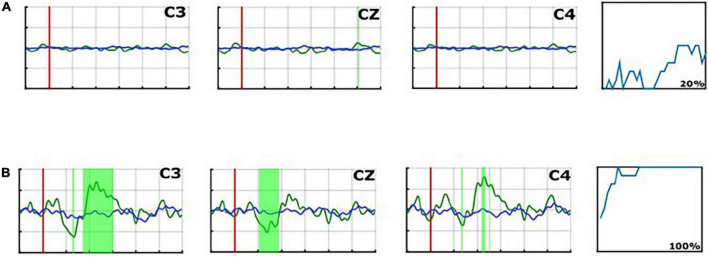
Average P300 ERP waveforms of the “C3,” “Cz,” and “C4” electrodes in run 1 for Patient 16 **(A)**, and for Patient 8 **(B)**. The red line presents the averaged non-target trials. The averaged target trials are plotted in blue. The magenta vertical line shows the trigger time. Green areas mark areas in which the target vs. non-target lines differ significantly.

**FIGURE 4:**
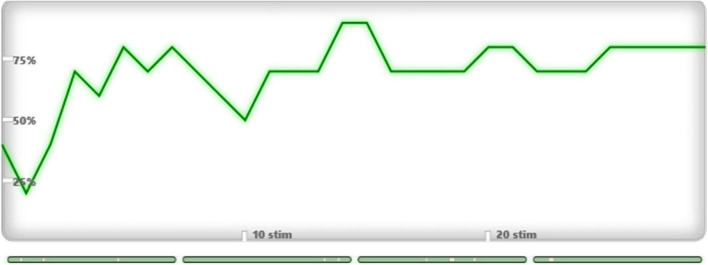
Plotted line graph of the rate of target stimulus detection for Patient 1, Week 1 (Accuracy: 70%).

**FIGURE 5:**
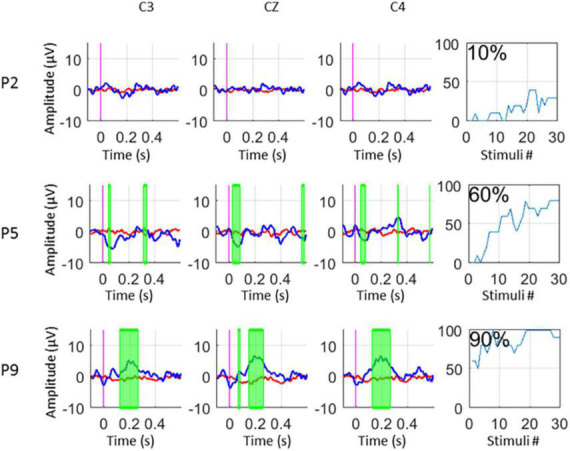
ERPs and Accuracy% for UWS (P2), MCS- (P5), and MCS + (P9) patients.

At the end of the data processing, the system calculated the median accuracy in %. Since the ratio of target to non-target stimuli is 1:7, chance accuracy was 12.5%. This accuracy indicates how well the system could discriminate target stimuli from other stimuli and may reflect each patient’s ability to follow instructions and count target stimuli (Figures 3A,B, 4, 5).

In addition to classification accuracy, we performed a Kruskal Wallis test (*p* < 0.05) to find statistical differences between target and non-target trials in the evoked potentials. A significance test is shown on the experimenter’s monitor, which presents areas with significant differences between targets and non-targets as green-shaded areas in the EPs (*p* < 0.05). Trials with an amplitude of the EEG signal exceeding ±100 μV are rejected from the EP and classifier calculation. The median of the excluded trials per session is only 9/480 (1,8%), IQR: 1–33 which shows the high data-quality of the recordings.

### Assessment withdrawal

Patient 13 interrupted the assessment at week 5 because of pulmonary infection with fever, respiratory failure and worsening of the overall clinical status. Patient 14 moved to another unit at week 6 to undergo endoscopic surgery. Patient 15 moved to another rehabilitation unit at week 6. Despite the early discontinuation of the assessment, for all these three patients, we could obtain the score at the CRS-R administered at the end of week 7. All the other patients maintained stable clinical conditions during the experimental period and performed regular weekly assessments.

## Results

Table 2 shows the results obtained from the CRS-R and BCI-based assessments, as well the final clinical diagnosis based on the repetitive CRS-R administration. We did not exclude any CRS-R or BCI-based assessment.

**TABLE 2:** Results from clinical and neurophysiological repetitive assessments.

PT	Early diagnosis		Sessions							Final diagnosis
				
			S 1	S 2	S 3	S 4	S 5	S 6	S 7	
1	UWS	CRS-R	7	7	8	**11**	10	**11**	**11**	MCS+
			(1,1,2 1,0,2)	(1,1,2 1,0,2)	(1,2,2 1,0,2)	(1,4,2 1,1,2)	(1,3,2 1,1,2)	(1,4,2 1,1,2)	(1,4,2 1,1,2)	
		BCI	**+**	**+**	**+**	**+**	**+**	−	**+**	
2	UWS	CRS-R	3	4	5	6	5	6	6	UWS
			(1,1,0, 0,0,1)	(1,1,1, 0,0,1)	(1,1,1 1,0,1)	(1,1,1 1,0,2)	(1,1,1 1,0,1)	(1,1,1 1,0,2)	(1,1,1 1,0,2)	
		BCI	−	−	−	−	**–**	**+**	**+**	
3	UWS	CRS-R	9	6	9	9	9	8	10	MCS-
			(2,1,2 2,0,2)	(1,1,1 1,0,2)	(2,1,2 2,0,2)	(2,1,2 2,0,2)	(2,1,2 2,0,2)	(2,1,2 1,0,2)	(2,2,2 2,0,2)	
		BCI	−	**+**	**+**	−	**+**	**+**	**+**	
4	UWS	CRS-R	6	9	9	9	12	**14**	**14**	MCS+
			(1,1,2 1,0,1)	(2,1,2 2,0,2)	(2,1,2 2,0,2)	(2,1,2 2,0,2)	(2,3,3 2,0,2)	(2,4,4 1,1,2)	(2,4,4 1,1,2)	
		BCI	**+**	**+**	−	−	**+**	−	−	
5	UWS	CRS-R	5	8	8	11	11	11	11	MCS-
			(1,1,1 1,0,1)	(2,1,2 1,0,2)	(2,1,2 1,0,2)	(2,3,2 2,0,2)	(2,3,2 2,0,2)	(2,3,2 2,0,2)	(2,3,2 2,0,2)	
		BCI	**+**	**+**	−	**+**	−	**+**	**+**	
6	UWS	CRS-R	1	1	5	6	7	6	6	UWS
			(0,0,0 0,0,1)	(0,0,0 0,0,1)	(1,0,2 1,0,1)	(2,0,2 1,0,1)	(1,1,2 1,0,2)	(2,0,2 1,0,1)	(2,0,2 1,0,1)	
		BCI	−	−	−	−	**+**	−	**+**	
7	UWS	CRS-R	3	3	4	6	6	6	6	UWS
			(1,0,0 1,0,1)	(1,0,0 1,0,1)	(1,0,0 1,0,2)	(1,0,2 1,0,2)	(1,0,2 1,0,2)	(1,0,2 1,0,2)	(1,0,2 1,0,2)	
		BCI	+	**+**	**+**	−	**+**	**+**	**+**	
8	MCS-	CRS-R	9	9	8	9	9	10	9	MCS+
			(1,2,2 2,0,2)	(1,2,2 2,0,2)	(2,1,2 1,0,2)	(1,2,2 2,0,2)	(1,2,2 2,0,2)	(2,2,2 2,0,2)	(1,2,2 2,0,2)	
		BCI	**+**	**+**	**+**	**+**	**+**	**+**	**+**	
9	UWS	CRS-R	5	6	6	10	10	10	10	MCS+
			(0,1,2 0,0,2)	(1,1,2 0,0,2)	(1,1,2 0,0,2)	(1,3,3 0,0,2)	(1,3,3 0,0,2)	(1,3,3 0,0,2)	(1,3,3 0,0,2)	
		BCI	**+**	**+**	**+**	**+**	**+**	**+**	**+**	
10	MCS-	CRS-R	11	8	8	8	10	10	8	MCS-
			(2,3,3, 1,0,2)	(1,2,2 1,0,2)	(1,2,2 1,0,2)	(2,2,2 1,0,1)	(2,3,2 1,0,2)	(2,3,2 1,0,2)	(2,2,2 1,0,1)	
		BCI	−	−	−		**+**	−	−	
11	UWS	CRS-R	6	6	8	8	10	10	**14**	MCS+
			(1,1,2, 1,0,1)	(1,1,2, 1,0,1)	(2,2,2, 1,0,1)	(2,2,2, 1,0,1)	(2,3,2 1,0,2)	(2,3,2 1,0,2)	(3,4,4 1,0,2)	
		BCI	**+**	−	**+**	−	−	−	−	
12	UWS	CRS-R	9	9	**14**	**16**	**16**	10	**15**	MCS+
			(1,2,2, 2,0,2)	(1,2,2, 2,0,2)	(3,4,1, 2,1,3)	(2,4,5, 2,1,2)	(2,4,5, 2,1,2)	(2,3,1, 1,1,2)	(2,3,5, 2,1,2)	
		BCI	**+**	**+**	**+**	**+**	**+**	**+**	**+**	
13	UWS	CRS-R	6	5	6	6				UWS
			(1,1,2, 1,0,1)	(1,1,1, 1,0,1)	(1,1,2, 1,0,1)	(1,1,2, 1,0,1)				
		BCI	**+**	**+**	−	**+**				
14	UWS	CRS-R	7	6	7	6	7			UWS
			(1,1,2, 2,0,1)	(1,1,2, 1,0,1)	(1,1,2, 2,0,1)	(1,1,2, 1,0,1)	(1,1,2, 2,0,1)			
		BCI	−	−	−	−	−			
5	UWS	CRS-R	7	7	7	6	6			UWS
			(1,1,2, 1,0,2)	(1,1,2, 1,0,2)	(1,1,2, 1,0,2)	(1,1,1, 1,0,2)	(1,1,2, 1,0,1)			
		BCI	−	−	−	−	−			
16	UWS	CRS-R	7	5	5	6	7	5	7	UWS
			(1,1,2, 1,0,2)	(0,0,2, 1,0,2)	(0,0,2, 1,0,2)	(1,1,2, 1,0,1)	(1,1,2, 1,0,2)	(1,0,2, 1,0,1)	(1,1,2, 1,0,2)	
		BCI	−	−	−	−	−	−	−	

For each patient, the “Command following” column refers to the detection of command following at the CRS-R or at the BCI paradigm. The CRS-R scores are reported in the same order of the assessment manual (auditory function; visual function; motor function; oromotor/verbal function; communication; arousal). The CRS-R assessments showing command following are marked in bold. The BCI assessments showing/not showing command following are signed with±. The “Final diagnosis” column reports the final clinical diagnosis resulted from the repetitive CRS-R assessment.

### Week 1 assessments

A total of 14 (1, 2, 3, 4, 5, 6, 7, 9, 11, 12, 13, 14, 15, 16) out of 16 patients did not show volitional behaviour at the CRS-R administration, suggesting a clinical diagnosis of UWS. Among these 14 patients, 8 (1, 4, 5, 7, 9, 11, 12, 13) attained a score of ≥ 50% at the first BCI-based assessment, indicating command following.

### Three-week analyses

At the end of the third week, Patient 3 attained accuracy above 50% in two BCI-based assessments, whereas the CRS-R still yielded no signs of willful behavioural responses.

Patient 12, who was responsive with the BCI from the first assessment, started to exhibit verbal command following at the third CRS-R administration. No changes in the clinical and neurophysiological responsivity were recorded with the other patients.

### Seven-week analyses

After seven CRS-R repetitions, seven patients with an initial diagnosis of UWS (1, 3, 4, 5, 9, 11, 12) showed volitional behaviours at least once at one or more CRS-R subscales, suggestive of MCS. All these patients started showing command following through the BCI paradigm from 2 to 6 weeks before the change in the clinical diagnosis was apparent *via* the CRS-R.

Four patients (2, 6, 7, 13) invariably obtained a CRS-R score leading to a UWS diagnosis but repeatedly attained BCI accuracy above 50%, indicating that the clinical and the BCI assessments suggested different diagnoses.

Three patients (14, 15, 16) did not exhibit command following through the CRS-R nor the BCI assessments, nor other signs of MCS.

The four patients who had a final diagnosis of MCS- (3, 5, 9, 10) were responsive in the BCI paradigms across multiple sessions, except Patient 10, who responded in only one session.

The five patients who had a final diagnosis of MCS+ (1, 4, 8, 11, 12) were also responsive in multiple BCI sessions.

## Discussion

Here, we compared two approaches to assess command following: a standardised clinical tool (the CRS-R) and a P300 BCI paradigm based on vibrotactile evoked potentials. For this purpose, sixteen patients were recruited at the time of the admission in the Rehabilitation Unit and assessed weekly for 7 weeks. The BCI data were collected blindly respect to the medical condition and the CRS-R data, as well as the prior diagnosis. We observed the appearance of command following revealed by the two methodologies across several sessions.

The experimental design aimed at overcoming the motor and cognitive limitations expected in this group of patients due to severe brain damage. Concerning the sensory stimuli modality, we used a visual-independent paradigm, considering the evidence collected from the clinical practice that most patients with DOC lack control of the neck and visual pursuit and attention.

Moreover, we opted for a somatosensory stimulation based on the note preservation of sensory perception abilities in UWS and MCS patients (Kassubek et al., 2003).

To prevent the effects of a possible short attention span, we applied an as fast as possible paradigm (about 2.5 min) without a preliminary test in the same session. We aimed with these measures to increase at most the participation of the patients in the BCI paradigm.

We used a paradigm in which the patient was asked to discriminate between two equally frequent stimuli (i.e., the vibration on the left or right wrist) while ignoring a more frequent distractor stimulus. This experimental paradigm is based on the three-stimulus oddball model (Squires et al., 1975; Polich, 2007). The instruction to count only the target stimulus – a stimulation on the target wrist – should elicit a robust P3b and other ERPs to that target stimulus only. This mental task entails both lower-level stimulus-driven mechanisms, also known as bottom-up processing, and higher-level attentional and other processes required for willful discrimination of persistent stimuli, or top-down processing.

The repetitive assessment with the two methodologies over 7 weeks obtained remarkable results. As previously reported (Wannez et al., 2017), the first CRS-R administration understated the behavioural responsivity of the patients, leading to an erroneous diagnosis of UWS for several patients. In the subsequent CRS-R repetitions, 7 out of 14 initially non-responsive patients started showing command following or other signs of volitional behaviour, and consequently changed their diagnoses in MCS. Six of them exhibited active command following during the BCI paradigm in the first session, demonstrating a high sensitivity to the command following of this neurophysiological approach.

Overall, we observed a strong relationship between the performance in the BCI paradigm and the clinical condition. All the MCS patients showed some responsivity in the BCI paradigm, including the MCS- patients, who had not otherwise shown any capacity to process language and execute commands. This evidence confirms the possible dissociation between cognitive abilities and observable behavioural phenomena, also known as covert consciousness.

All patients who showed command following behaviourally were able to achieve high accuracy scores repeatedly. Interestingly, four of sixteen patients, clinically judged as UWS, were able to follow commands *via* the BCI paradigm even though CRS-R assessments conveyed insufficient behavioural signs of awareness. This result confirms previous evidence collected with the same paradigm (Guger et al., 2018; Spataro et al., 2018) and other methodologies (Bodart et al., 2017) and strengthens the need for an integrated approach to the evaluation of consciousness. The repeated assessments also showed that the BCI approach might detect covert command following before the behavioural observation.

These results extend our previous report (Spataro et al., 2018), in which we used the same approach to demonstrate that somatosensory discrimination could help predict patients’ recovery of behavioural responsivity. Advance detection of minimal responsivity might be crucial in clinical contexts in which several repetitions of the CRS-R are hard to perform (e.g., due to difficulties in interrupting the sedation, when needed) or when the patient’s ability to respond might influence decisions on life-prolonging interventions or end-of-life decisions. Furthermore, if patients can potentially communicate through a BCI or similar communication system, they might make their own decisions.

The repetitive assessment showed fluctuations in the responsivity at both the behavioural and BCI paradigms. Variable response to stimulations in the same patient is a characteristic feature of the DOC (Schnakers et al., 2009) and motivates the need for repetitive CRS-R administrations to improve diagnostic precision (Wannez et al., 2017). Similarly, the BCI assessment requires sustained attention for the duration of the experiment, notwithstanding several circumstances potentially interfering with the accomplishment of the mental task (e.g., sleepiness, discomfort, noises, etc.). This evidence explains low accuracy scores repeatedly collected even in behaviourally responsive patients (MCS+) and paves the way for future studies on the sensitivity and specificity of the present paradigm. However, these disease-related limitations do not diminish the primary significance of the proposed diagnostic tool, which revealed command following in patients judged as unresponsive at the bedside.

Hence, the integration of active BCI-based paradigms in the clinical diagnosis of the DOCs might help confirm or challenge a clinical judgement of unresponsiveness based on behavioural assessments, providing reproducible proof of voluntary mental processes. This information may support decisions regarding life-sustaining therapies, intensive rehabilitation programs, treatment of pain, and enhance the interaction of family and friends with the patient. With further research, other BCI paradigms that detect other ERPs such as the N400 or mismatch negativity (MMN), explore activity from emotion, and/or employ non-EEG signals could not only improve diagnostic accuracy and prediction but also provide additional details about each patients’ remaining abilities and support treatment (Erlbeck et al., 2017).

Given the relatively low cost and ease of assessing patients at the bedside with EEG BCI systems and the substantial absence of contraindications, this approach might improve the diagnosis and management of the DOC on a large scale, including small or non-specialised clinical centres.

Future studies will involve larger cohorts of DOC patients, to be assessed with different methods [clinical judgement call, multiple (≥ 5 per period) repetitive CRS-R administration, BCI paradigms], to investigate the sensitivity and specificity of the single approaches. Moreover, a prolonged follow-up (3/6/12 months) will provide information on the putative prognostic value of the behavioural and neurophysiological signs of responsivity collected.

## Limitations

This study is limited by the recruitment of a relatively small sample of patients, and the early withdrawal of three of them. We also included patients with different etiologies of DOC and different disease durations, and hence could not explore differences in the BCI performance related to these factors. Future studies are needed to study larger patient groups in more detail and with more sessions where possible. Such studies will take time, due mainly to the emphasis on patient factors (including safety, health, comfort, and choice) as well as the challenges of conducting many sessions that require a trained assessor.

We blindly observed the appearance of command following at the behavioural assessment and the BCI paradigm without knowledge of the clinical condition. Moreover, the responsiveness to active paradigms may be compromised by aphasia, which may partially or totally impair comprehension of verbal instructions. Future studies will examine the relationship between the structural damage of specific neuronal pathways, (such as the frontal-parieto-temporal circuits engaged in attentional processes, the somatosensory pathways and others) and the evidence of overt and covert command following.

This study is also limited by the paradigms used to elicit ERP activity for classification. Other studies have employed other stimuli, task instructions, and paradigms that might elicit different ERPs, which might lead to improved classification and/or additional detail about each patient’s remaining cognitive functions and prognosis. In addition, paradigms that might yield more robust target vs. non-target differences - and/or use activity from the N2, N4, MMN, other ERPs, and/or non-ERP information - could improve diagnostic and/or predictive accuracy and detail (Squires et al., 1975; Fischer et al., 2008; Balconi et al., 2013; Erlbeck et al., 2017; Pan et al., 2018). Finally, we did not apply multiple testing correction when assessing command following, and different analysis methods or accuracy thresholds may produce different results. Future work should explore which combinations of paradigms, parameters and analyses are most effective, both for initial assessment and then more detailed evaluation.

## Conclusion

Brain-Computer Interface paradigms that do not require patients to move nor see might improve clinical evaluation of DOC patients by (1) increasing diagnostic accuracy by detecting command following when behavioural assessments fail and (2) facilitating outcome prediction by anticipating the emergence of minimal consciousness. Multiple sessions are recommended to provide accurate assessment.

## Data availability statement

The raw data supporting the conclusions of this article will be made available by the authors, without undue reservation.

## Ethics statement

The studies involving human participants were reviewed and approved by the Comitato Etico IRCCS Centro Neurolesi Bonino Pulejo, Messina, Italy. Written informed consent to participate in this study was provided by the participants’ legal guardian/next of kin. Written informed consent was obtained from the individual(s) for the publication of any potentially identifiable images or data included in this article.

## Author contributions

RS designed the study, collected the clinical data, performed data analysis and interpretation, and drafted and revised the manuscript. YX performed the BCI assessments. RX collaborated in the manuscript draft (methodological section) and revision. GM helped in clinical data acquirement. AH collaborated in BCI data collection. BA contributed to the scientific writing and revision of the drafted manuscript. VL reviewed the drafted manuscript. CG provided the equipment used in this research and collaborated in the study design. All authors contributed to the article and approved the submitted version.
